# Immune interception in cancer: prioritizing vaccination in high-risk and premalignant settings

**DOI:** 10.3389/fimmu.2026.1862551

**Published:** 2026-05-15

**Authors:** Quanbin Zhang, Le Wang, Liping Ze, Tao Wei, Wenpeng Zhang

**Affiliations:** 1 Department of Traumatology and Orthopaedics, Zibo Central Hospital, Zibo, China; 2 Department of General Practice, Zibo Central Hospital, Zibo, China; 3 Department of Joint and Sports Medicine, Zibo Central Hospital, Zibo, China

**Keywords:** cancer prevention, cancer vaccines, immune interception, minimal residual disease, neoantigens

## 1 Introduction

Over the past three decades, the field of cancer vaccines has been marked by both enthusiasm and disappointment (1, 2). While numerous vaccine platforms—from peptide and dendritic cell formulations to viral vectors and mRNA constructs—have demonstrated safety and immunogenicity, their clinical impact in advanced disease has often been modest (3, 4). The prevailing challenge is that late-stage cancers exist in a profoundly immunosuppressive environment, where regulatory T cells, myeloid-derived suppressor cells, and exhausted effector T cells dominate (5). In such settings, even the most sophisticated vaccines struggle to elicit clinically meaningful responses. This reality has prompted a critical reevaluation of the conventional approach: instead of trying to reverse entrenched immune dysfunction in bulky, late-stage disease, perhaps the real opportunity lies in moving vaccination upstream, when the immune system is more intact and the tumor burden is minimal or even pre-clinical (6).

The rationale for upstream vaccination is compelling. In high-risk individuals—such as those with hereditary cancer syndromes, precancerous lesions, or minimal residual disease after surgical resection—the immunological landscape is significantly less hostile (7). Antigens are more stable and predictable, ranging from recurrent driver mutations like *KRAS* or *TERT* promoter alterations to tumor-associated proteins aberrantly expressed during early carcinogenesis (8). The safety threshold is also more manageable, as vaccines can be deployed before widespread tissue invasion or systemic debilitation. Importantly, the potential impact extends beyond incremental benefit: vaccines in these settings could truly intercept cancer before it becomes clinically established, shifting the paradigm from treatment to prevention (9). This concept of “immune interception” reframes cancer vaccination as a proactive measure, aligning it with public health strategies while still leveraging the precision of modern immunology. In addition, central nervous system (CNS) tumors such as glioblastoma exemplify the extreme challenge of late-stage vaccination, as the blood–brain barrier and profound local immunosuppression further hinder therapeutic efficacy (10, 11). Building on this rationale, it becomes essential to examine not only the theoretical advantages of upstream vaccination but also the growing body of clinical evidence that supports its feasibility and promise. In this Opinion, we distinguish between evidence-supported observations and forward-looking hypotheses. Current human data most strongly support the safety, feasibility, and immunogenicity of upstream cancer vaccination, whereas claims regarding durable cancer prevention, population-level benefit, and regulatory approval remain provisional and require validation in adequately powered randomized trials with clinically meaningful endpoints (12).

## 2 Why upstream/high-risk/premalignant is a strong strategy

The case for prioritizing vaccination in upstream settings rests on several interlocking advantages. First, the immunological terrain is far more favorable before a tumor fully establishes itself. In high-risk or premalignant states, the density of regulatory T cells, myeloid suppressor cells, and inhibitory cytokines is lower, allowing vaccines to engage dendritic cells and T lymphocytes more effectively (13). This contrasts with advanced cancers, where immune checkpoints and metabolic constraints blunt vaccine-induced responses. Second, antigen predictability is higher in these contexts (14, 15). Germline syndromes such as Lynch syndrome can generate recurrent, well-characterized neoantigenic targets, while shared driver alterations such as KRAS and, in selected tumor types, TERT promoter mutations may provide broader vaccine targets (16). Such stability enables both personalized and off-the-shelf formulations, reducing the problem of antigen escape (17).

A third advantage is safety and tolerability. Deploying vaccines in otherwise healthy but high-risk individuals necessitates a high safety margin, yet early data from minimal residual disease and prevention trials suggest that modern formulations—amphiphile vaccines, long peptides with safe adjuvants, or viral vectors combined with immune modulators—can meet this standard without triggering severe autoimmunity (18, 19). Finally, the public health impact of upstream vaccination could be transformative. Importantly, the principle that vaccination can prevent cancer is already established in virus-associated malignancies. Prophylactic HPV vaccination has been associated with substantial reductions in cervical cancer incidence at the population level, and universal hepatitis B vaccination in Taiwan was followed by a marked decline in childhood hepatocellular carcinoma (20). These examples do not prove that non-viral cancer vaccines will achieve similar outcomes, because viral antigens are more foreign and immunologically distinct than most tumor-associated or self-derived antigens. Nevertheless, they provide a public-health precedent for cancer prevention through immune priming and support the broader conceptual basis of immune interception. In the long term, if efficacy and durability are confirmed, upstream cancer vaccines could potentially reduce cancer incidence, lower treatment-related costs, and improve population-level survival. At present, however, this remains a forward-looking hypothesis rather than an established clinical outcome for non-viral cancer vaccines (21). Taken together, these considerations illustrate why the strategic shift from treatment to interception is biologically plausible and clinically worth testing, rather than already established as a standard approach. This rationale sets the stage for evaluating recent clinical progress that supports the feasibility, but not yet the definitive efficacy, of upstream cancer vaccination. This rationale may also conceptually extend to neuro-oncology, where inherited cancer predisposition syndromes such as Li-Fraumeni syndrome highlight the possibility that future interception strategies could be explored before glioma becomes clinically apparent (22).

**Figure 1:**
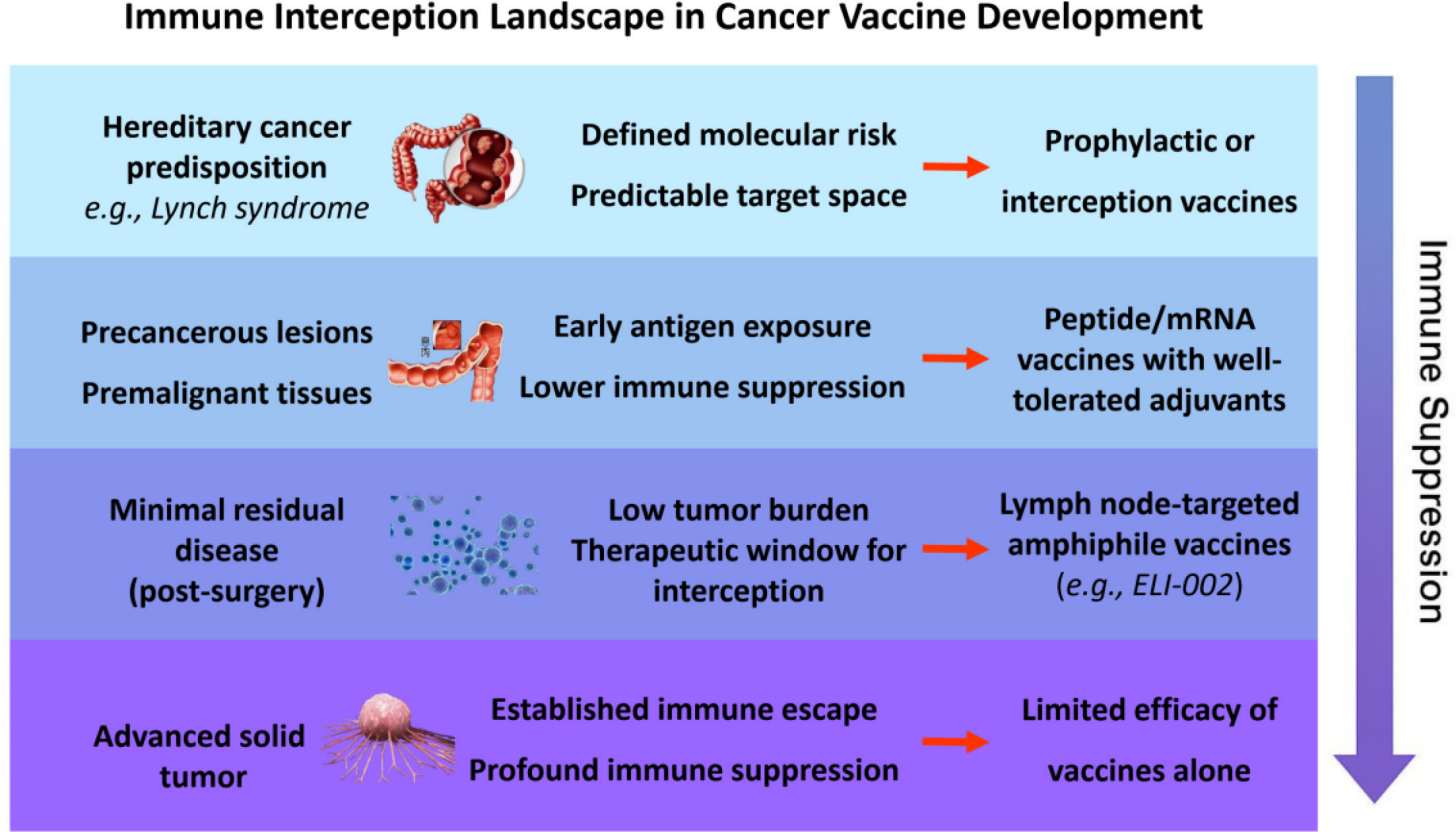
Immune interception landscape in cancer vaccine development. As cancer progresses from hereditary risk and premalignant lesions to minimal residual disease and advanced solid tumors, immune suppression generally increases. Upstream settings may provide more favorable conditions for cancer vaccination, including lower tumor burden, more predictable target biology, and less entrenched immune suppression, whereas vaccines alone are more likely to have limited efficacy in advanced solid tumors.

**Figure 1** illustrates the continuum of cancer progression from germline predisposition to advanced disease, highlighting the shifting immunological landscape and antigenic context. It emphasizes why upstream settings—such as genetic risk carriers, precancerous lesions, and post-surgical minimal residual disease—offer a more favorable terrain for vaccination, both immunologically and logistically. The figure also maps current vaccine strategies to their optimal intervention windows, reinforcing the rationale for shifting the cancer vaccine paradigm toward immune interception.

## 3 Emerging human evidence for upstream cancer vaccination

Several recent trials support the feasibility, safety, and immunogenicity of vaccinating earlier in the cancer trajectory. However, the strength of evidence differs across endpoints. Safety and vaccine-induced immune activation are directly supported by early-phase human data; associations with delayed relapse or molecular response are suggestive but not definitive; and claims of durable cancer prevention or population-level incidence reduction remain hypothetical until confirmed in randomized trials with long-term follow-up.

ELI-002 2P (KRAS-targeted, lymph node-targeting amphiphile vaccine): In a phase I study in pancreatic and colorectal cancer patients with mutant KRAS after standard locoregional therapy, thus in a high-risk relapse/minimal residual disease (MRD) setting, ELI-002 (NCT04853017) 2P induced robust mutant KRAS-specific CD4+ and CD8+ T cell responses in most patients, with many responses persisting over time (23, 24). Importantly, updated long-term follow-up from the AMPLIFY-201 study further strengthened the signal that higher vaccine-induced T cell responses were associated with more favorable relapse-related outcomes, supporting the concept that vaccination may be most effective when tumor burden is low and immune suppression is less entrenched. Safety was favorable, with no cytokine release syndrome and no major late-emerging tolerability concerns reported. This remains one of the clearest human proof-of-concept examples supporting upstream vaccination in a molecularly defined postoperative/MRD context (25, 26). Nevertheless, these data should be interpreted as early proof-of-concept evidence. The observed association between stronger T cell responses and favorable relapse-related outcomes supports further testing, but it does not by itself establish recurrence prevention.
Tri-Ad5 + N-803 in Lynch syndrome (NCT05419011): This remains one of the more advanced prevention-oriented vaccine efforts. In patients with Lynch syndrome, who carry high lifetime risks of colorectal, endometrial, and other cancers, an ongoing randomized phase IIb trial is evaluating a multivalent adenovirus-5 vaccine targeting CEA, MUC1, and brachyury in combination with the IL-15 superagonist N-803, with the goal of reducing colorectal neoplasia. While this program has not yet generated the same level of published efficacy signal as ELI-002, it is important because it shows that vaccine-based interception strategies are being tested not only after cancer treatment, but also in genetically high-risk individuals before invasive cancer develops. Early/open-label phases have completed accrual, underscoring the practical feasibility of this prevention-oriented platform (27). Earlier MSI-H colorectal cancer vaccine studies, such as the frameshift peptide vaccine trial NCT01461148, helped establish the clinical feasibility and immunogenicity of shared MSI-associated neoantigen targeting, although these studies were conducted in established colorectal cancer rather than in a true prevention or interception setting. Therefore, this program should be presented as a prevention-oriented trial platform rather than as evidence that vaccination has already reduced cancer incidence in Lynch syndrome.
Mutant KRAS long peptide vaccine (NCT05013216, phase I): Testing the safety and immunogenicity of a pooled mutant KRAS-targeted long peptide vaccine with the adjuvant poly-ICLC, this line of investigation is especially relevant because it extends the field beyond tumor-associated antigens toward shared driver-mutation vaccination (28). Although not strictly preventive in all enrolled populations, it helps establish the clinical tractability of driver-directed vaccine design and the use of potent innate immune adjuvants in settings that are biologically upstream relative to widely metastatic disease. More recent KRAS vaccine studies have also continued to support the broader principle that mutant KRAS can serve as a clinically actionable immunologic target in earlier-stage or high-risk settings (29, 30). Thus, the main implication of these studies is target tractability and immune feasibility, not yet proof of broad preventive efficacy.
Nous-209 vaccine in Lynch syndrome (NCT05078866): This example can now be stated more strongly than before. Rather than being described only as a trial aiming to assess safety, Nous-209 has now generated published phase 1b/2 data in Lynch syndrome carriers, showing acceptable safety together with measurable vaccine-specific T cell induction, including breadth and durability of responses (31). This is particularly important because it moves the field closer to a true interception/prevention setting in genetically predisposed individuals, rather than only postoperative relapse prevention (17). Accordingly, Nous-209 now represents one of the strongest available human examples that upstream cancer vaccination is feasible in individuals without advanced malignancy. Importantly, these findings support immune feasibility in genetically predisposed individuals, but longer follow-up is still needed to determine whether vaccine-induced immunity translates into reduced adenoma burden, cancer incidence, or cancer-related mortality.

These studies collectively show: (i) vaccines targeting shared driver or tumor-associated antigens can elicit measurable and sometimes durable immune responses in humans; (ii) upstream settings, including postoperative MRD and hereditary cancer predisposition, are clinically feasible and generally compatible with the safety expectations of prevention-oriented development; and (iii) there is now emerging human evidence, strongest for ELI-002 and increasingly supported by Lynch syndrome vaccine programs such as Nous-209, that immune activation in these settings may have clinically meaningful translational relevance. At the same time, it should remain explicit that most available studies are still early-phase, and definitive randomized evidence for broad cancer-incidence reduction or recurrence prevention remains limited.

## 4 Challenges and strategic directions

Despite encouraging progress, the pathway to establishing upstream cancer vaccines as a standard intervention faces significant hurdles. These challenges, however, also illuminate the directions in which the field must strategically evolve. These hurdles are not only theoretical. Earlier therapeutic cancer vaccine programs illustrate how biological and methodological barriers can weaken otherwise rational strategies. For example, single-antigen or narrow-epitope vaccines have been vulnerable to heterogeneous antigen expression, HLA restriction, antigen loss, and immune escape. The MAGE-A3 program in resected melanoma and non-small-cell lung cancer is a cautionary example: despite a strong biological rationale and large phase III trials, adjuvant MAGE-A3 immunotherapy failed to improve disease-free survival compared with placebo, highlighting the difficulty of translating antigen-specific immune responses into clinical benefit in heterogeneous tumors (32). Similarly, many advanced-disease vaccine trials have been limited by heterogeneous patient populations, variable tumor burden, prior therapies, delayed kinetics of vaccine-induced immunity, and endpoints that may not fully capture immunologic benefit. These experiences support the need for multivalent antigen design, better biomarker-defined populations, and trial endpoints aligned with vaccine biology.

### 4.1 Immune tolerance and safety concerns

A primary barrier is the risk of autoimmunity when targeting tumor-associated antigens (TAAs) that are also expressed at low levels in normal tissues (33). In preventive contexts, where vaccines may be administered to otherwise healthy individuals, the safety bar is particularly high. Even low-frequency severe adverse events may be unacceptable if the vaccinated population has a relatively low absolute short-term cancer risk. Therefore, prevention-oriented vaccine development should begin in populations with clearly elevated risk, measurable premalignant or molecular endpoints, and strong biological rationale, rather than in broadly defined healthy populations. Strategic solutions include prioritizing driver mutations and neoantigens that are absent from healthy tissues, as well as employing vaccine delivery systems that restrict antigen presentation to professional antigen-presenting cells in lymphoid tissues. Early data from KRAS and Lynch syndrome vaccine trials suggest that this approach can generate potent responses without unacceptable toxicity (34).

### 4.2 Identifying the right populations

Another challenge lies in defining who should receive these vaccines (35). Beyond hereditary syndromes and MRD, future interception strategies may also be considered in other risk-defined populations. These include individuals with persistent oncogenic viral infection or virus-driven premalignant disease, patients with chronic inflammatory conditions that increase cancer risk, and people with well-documented carcinogen exposure, such as tobacco-related field cancerization or occupational/environmental exposure histories. However, these broader applications require more stringent risk stratification than hereditary syndromes, because the absolute cancer risk is often more heterogeneous and less predictable. For this reason, candidate populations should be selected using combinations of exposure history, molecular biomarkers, premalignant lesion status, viral persistence, immune profiling, and longitudinal risk models rather than exposure history alone (36). Strategic progress requires the integration of circulating tumor DNA (ctDNA), advanced imaging, and multi-omic risk models to identify individuals most likely to benefit, thereby balancing efficacy with ethical concerns of overtreatment (37, 38). In neurological oncology, integrating advanced imaging modalities such as MRI with ctDNA could help stratify individuals at risk of early glioma, thereby defining a population suitable for preventive vaccination trials.

### 4.3 Antigenic heterogeneity and immune escape

Even in early lesions, tumors can evolve to escape immune pressure by losing or downregulating target antigens. Vaccines directed against single epitopes are especially vulnerable to this limitation (39). Previous single-antigen strategies have shown that target expression at baseline does not guarantee durable clinical control, because antigen expression may be spatially heterogeneous, restricted to subclones, or lost under immune selection. In addition, HLA restriction can narrow the eligible population and reduce the generalizability of peptide-based vaccines. These limitations help explain why multivalent, modular, or personalized vaccine designs are increasingly favored over single-antigen approaches (40). A promising direction is the development of multivalent or modular vaccines, combining shared driver mutations with panels of well-characterized TAAs, thereby broadening coverage and reducing escape potential (41). Advances in mRNA technology and synthetic long peptides now allow for rapid customization and iteration of such multicomponent formulations.

### 4.4 Clinical trial design and regulatory pathways

Prevention trials require long follow-up periods and large cohorts to demonstrate reductions in cancer incidence, creating logistical and financial obstacles. Moreover, regulatory frameworks for preventive cancer vaccines remain underdeveloped. In prevention-oriented settings, the challenge is different but equally demanding: cancer incidence and cancer-specific mortality are definitive but slow endpoints, whereas immune response, ctDNA clearance, adenoma recurrence, or premalignant lesion regression remain candidate surrogate endpoints that require validation. Regulatory uncertainty therefore remains substantial, because immune response alone is unlikely to be sufficient for approval unless it is linked to a clinically meaningful outcome. To address these issues, innovative trial designs are essential—using adaptive endpoints such as immune response magnitude, ctDNA clearance, or adenoma recurrence as validated surrogates (42). In parallel, early dialogue with regulators and health agencies is needed to establish acceptable safety and efficacy thresholds for preventive indications.

### 4.5 Integration with immunomodulatory strategies

Finally, vaccines alone may not suffice, even upstream. Combining vaccination with checkpoint inhibitors, metabolic modulators, or IL-15 superagonists could amplify responses without requiring prolonged therapy (43, 44). These rational combinations should be carefully tailored to early-disease contexts, where transient and less toxic interventions may be sufficient to achieve durable protection.

Taken together, these challenges underscore the complexity of implementing upstream vaccination. Yet, each obstacle points directly toward a strategic solution, suggesting that the field already has the conceptual and technological tools required to transform immune interception into a viable clinical reality.

## 5 Toward a preventive paradigm in cancer immunology

The trajectory of cancer vaccine research increasingly suggests that their greatest value will not be realized in advanced disease but in preventive and interception settings. Within the next decade, several pivotal shifts are foreseeable (45). Vaccines targeting recurrent driver mutations such as *KRAS* or frameshift neoantigens in mismatch repair deficiency may become candidates for future regulatory evaluation as non-viral cancer prevention or interception vaccines, particularly in high-risk cohorts such as Lynch syndrome carriers. However, such approval will depend on durable safety data, validated surrogate endpoints, and evidence that immune responses translate into reduced cancer incidence or recurrence (46). In parallel, the integration of minimal residual disease monitoring through circulating tumor DNA could enable adaptive deployment of vaccines after surgery, establishing immune surveillance as a complement to traditional adjuvant therapies. This preventive paradigm should not be interpreted as indiscriminate vaccination of healthy populations. Rather, its near-term application is most defensible in settings where risk is high, target biology is well defined, and clinical or molecular endpoints can be monitored. These include hereditary cancer syndromes, persistent oncogenic viral infection, premalignant lesions, and MRD-positive postoperative states. Broader use in environmentally exposed populations will require stronger predictive biomarkers and careful demonstration that expected benefit outweighs potential harm.

Technological advances—particularly modular mRNA platforms and lymph node–targeted delivery systems—will accelerate the translation of multivalent vaccines into clinical reality, allowing flexible combinations of shared and individualized antigens (47). At the policy level, engagement with regulators and health agencies will be critical to define safety thresholds and surrogate endpoints suitable for prevention trials, thereby shortening the path from immunogenicity to licensure.

Cost-effectiveness and global health equity should also be incorporated into the development of preventive cancer vaccines. If upstream vaccination can prevent recurrence or malignant transformation, it may reduce the long-term economic burden associated with surgery, systemic therapy, surveillance, and end-of-life care. However, this potential benefit must be balanced against the high upfront costs of vaccine manufacturing, biomarker-based risk stratification, immune monitoring, and long-term follow-up. This concern is particularly relevant for individualized neoantigen vaccines and complex delivery platforms, which may be difficult to scale in low- and middle-income settings. Lessons from HPV vaccination show that population-level cancer prevention requires not only biological efficacy, but also affordable pricing, simplified delivery, broad coverage, and equitable access. Therefore, future development should prioritize scalable platforms, cost-effectiveness analyses, and implementation strategies that prevent upstream cancer vaccination from widening existing disparities in cancer prevention and care (48).

The overarching vision is a shift from reactive treatment to proactive immune interception: identifying those at greatest risk, vaccinating before immune suppression takes hold, and maintaining durable protection against malignant progression (49). Such a paradigm could potentially reduce cancer incidence and mortality and reframe oncology as a discipline where prevention stands alongside therapy as a central pillar. However, important uncertainties remain regarding long-term efficacy, durability of immune protection, late-emerging safety signals, optimal booster strategies, and the balance between benefit and risk in healthy or asymptomatic high-risk individuals. The progress of the past few years suggests that this vision is no longer purely aspirational, but its realization will require long-term follow-up, rigorous safety surveillance, validated clinical endpoints, and transparent risk–benefit assessment.
